# Preoperative Botulinum Toxin for Complex Diaphragmatic Paralysis: A Case Series

**DOI:** 10.3389/jaws.2025.14476

**Published:** 2026-01-07

**Authors:** L. Sánchez Moreno, Z. Valera Sánchez, J. R. Naranjo Fernández, S. Morales-Conde

**Affiliations:** Department of General and Digestive Surgery, University Hospital Virgen Macarena, Seville, Spain

**Keywords:** botulinum toxin type A, compartment syndrome, diaphragmatic paralysis, prehabilitation, surgical treatment

## Abstract

**Introduction:**

The management of giant diaphragmatic paralysis remains a significant surgical challenge, frequently associated with high rates of recurrence and the risk of developing abdominal compartment syndrome. While the use of Botulinum Toxin Type A (BTX- A) as an adjuvant therapy has been established in complex ventral hernia repair, its application in diaphragmatic paralysis is novel and sparsely documented. This study aims to present our institutional experience with BTX-A as a prehabilitation strategy in patients with complex diaphragmatic paralysis and to evaluate short- and long-term outcomes.

**Materials and Methods:**

Three patients with complex diaphragmatic paralysis underwent preoperative administration as part of a prehabilitation protocol prior to surgical repair. Loss of domain (LD) was calculated using the Sabbagh formula. According to Sabbagh, LD is defined as the ratio of herniated volume to total peritoneal volume (LD = HV/TPV), with a loss >20% being considered significant. All patients received a standardized BTX-A administration protocol consisting of ultrasound-guided injection of 500 units of botulinum toxin type A, administered at six sites following the technique described by Smoot, with three injection points on each side targeting the internal oblique muscle 4 weeks before surgery.

**Results:**

Preoperative administration of Botulinum Toxin Type A (BTX-A) was safe in all three patients, with no postoperative complications or development of abdominal compartment syndrome, which was monitored through continuous intra-abdominal pressure measurements during the hospital stay (short-term outcomes). Six months postoperatively, all patients demonstrated significant improvement in respiratory function, assessed by standard pulmonary function tests, and reported improved quality of life, including relief from dyspnoea and enhanced daily functioning. At twelve months, two patients remained asymptomatic, with no clinical or radiological evidence of recurrence (long-term outcomes). Overall, preoperative BTX-A was associated with both short-term safety and sustained long-term functional benefits in this series.

**Conclusion:**

Preoperative BTX-A appears to be safe and well-tolerated in complex diaphragmatic paralysis. The results suggest that BTX-A may reduce complications, improve functional outcomes, enhance respiratory function, and increase quality of life, with effects maintained for at least 1 year in most patients.

## Introduction

Diaphragmatic eventration is a congenital or acquired condition characterized by abnormal elevation of the diaphragm due to muscular or phrenic nerve dysfunction while maintaining its continuity and attachment to the costal margins [1]. Phrenic paralysis, often secondary to nerve injury, thoracic or cervical surgery, trauma, or neurological disease, represents the most frequent functional disorder of the diaphragm [2, 3]. Symptomatic patients may present with dyspnoea, orthopnoea, hypoxemia, and nonspecific gastrointestinal complaints.

Surgical treatment, particularly diaphragmatic plication, remains the mainstay of therapy for symptomatic patients, aiming to improve respiratory function and relieve dyspnoea [1, 2]. However, giant or complex diaphragmatic paralysis poses significant challenges, with high recurrence rates and risk of abdominal compartment syndrome [4].

Botulinum Toxin Type A (BTX-A) has been increasingly used in complex ventral hernia repair. By inducing temporary flaccid paralysis of abdominal wall muscles, BTX-A facilitates fascial closure and reduces tension [5–12]. Its application in diaphragmatic paralysis remains scarcely documented. This study presents our experience using BTX-A as a prehabilitation strategy prior to diaphragmatic plication.

## Case Report

### Case 1

A 44-year-old man (body mass index 39 kg/m^2^) was evaluated for chest pain and dyspnoea with a two-year history. After assessment by the pulmonology, cardiology, and neurology departments, a diagnosis of idiopathic left diaphragmatic relaxation was made, and the patient was referred for thoracic surgery. His medical history was notable for suspected obstructive sleep apnoea syndrome (OSAS), managed with non-invasive mechanical ventilation (NIV). Physical examination was unremarkable.

**FIGURE 1:**
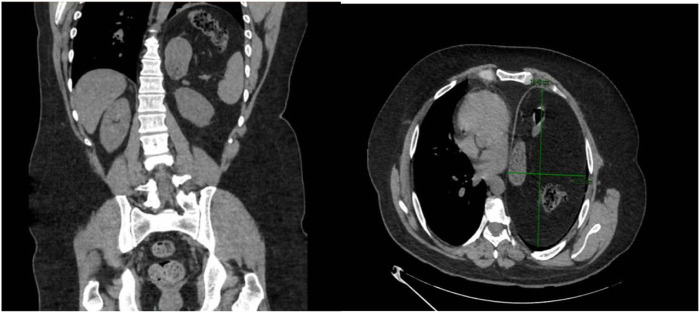
Computed tomography scan (coronal and transverse section). Significant elevation of the left hemidiaphragm and infradiaphragmatic abdominal contents, including the stomach, spleen, and splenic flexure of the colon and deviation of the mediastinum to the right.

A neurophysiological study and thoracic computed tomography scan (Figure 1) revealed a serious elevation of the left hemidiaphragm and herniation of abdominal contents into the thorax, including stomach, spleen, and colon splenic flexure, occupying approximately 50% of the left hemithorax and causing right mediastinal shift, with 20% of abdominal viscera displaced into the thorax according to Sabagg’s formula [13]. Preoperative preparation focused on prehabilitation with respiratory physiotherapy.

Given the size of the defect, adjuvant therapy was performed with botulinum toxin injection following the Smoot technique [14], without complications.

Four weeks later, a left diaphragmatic plication was performed via posterolateral thoracotomy, using non-absorbable monofilament barbed sutures and reinforced with a Gore- Tex® mesh. Intraoperative findings confirmed significant diaphragmatic laxity, with the dome of the diaphragm reaching the level of the bronchial carina. A pleural drain and an epidural catheter were placed for postoperative management.

The patient was extubated in the operating room and transferred to the postoperative recovery unit, where he remained for 4 days, hemodynamically stable, eupnoeic, and without need for supplemental oxygen. Intra-abdominal pressure was monitored, with values of 10 mmHg preoperatively, 11 mmHg at the end of surgery, and less than 15 mmHg over the subsequent days. The epidural catheter was removed at 72 h, the pleural drain removed on postoperative day 5, and the patient was discharged on day 6.

**FIGURE 2:**
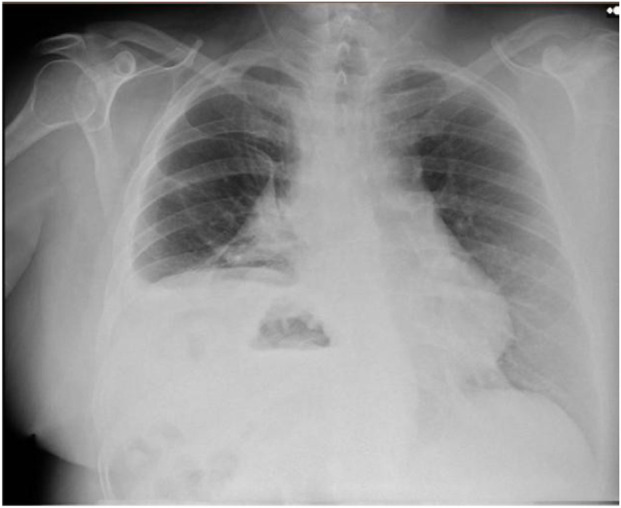
Postoperative Chest X-ray showed no elevation of the left hemidiaphragm.

At the six-month follow-up, the patient reported significant improvement in quality of life, including the ability to tolerate the supine position, which had previously been intolerable. He demonstrated good respiratory function, and a follow-up chest radiograph (Figure 2) showed no elevation of the left hemidiaphragm. At twelve months, the patient remained asymptomatic and recurrence-free.

### Case 2

A 63-year-old woman (body mass index 35 kg/m^2^) was evaluated for dyspnoea and digestive symptoms of 2 years’ duration. After being evaluated by the pulmonology department, idiopathic left diaphragmatic relaxation was diagnosed, and the patient was referred for thoracic surgery. Her medical history included being a former smoker for 20 years, and she was diagnosed with severe OSAHS (obstructive sleep apnoea-hypopnea syndrome). The physical examination was normal.

**FIGURE 3:**
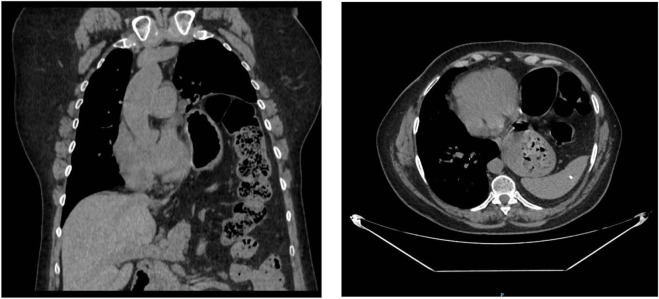
Computed tomography (coronal and transverse section) marked elevation of the left hemidiaphragm and herniation of abdominal contents into the thoracic cavity, including the stomach, spleen, and splenic flexure of the colon with a moderate contralateral mediastinal shift.

A chest CT (Figure 3) scan revealed a severe elevation of the left hemidiaphragm and herniation of abdominal contents into the thorax, including stomach, spleen, and colon splenic flexure, with a percentage of herniated viscera of 23% according to Sabagg’s formula [13], which occupied more than half of the left hemithorax. It is associated with compressive atelectasis and a moderate contralateral mediastinal shift. No image of a diaphragmatic defect was observed. Preoperative preparation focused on prehabilitation with chest physiotherapy. Given the size of the defect, adjuvant therapy was performed with botulinum toxin injection following the Smoot technique [14], without complications.

Four weeks later, a left diaphragmatic plication was performed via posterolateral thoracotomy, using nonabsorbable monofilament barbed sutures reinforced with Gore- Tex® mesh. Intraoperative findings confirmed significant diaphragmatic laxity, with the dome of the diaphragm reaching the main carina. A pleural drain and an epidural catheter were placed for postoperative management.

The patient was extubated in the operating room and transferred to the Postoperative Recovery Unit, where she remained hemodynamically stable, eupnoeic, and without the need for supplemental oxygen for 2 days. Intra-abdominal pressure was monitored, with values of 12 mmHg preoperatively, 12 mmHg at the end of surgery, and less than 15 mmHg in the following days. The epidural catheter was removed 72 h later, the pleural drain was removed on the fourth postoperative day, and the patient was discharged on the fifth day.

**FIGURE 4:**
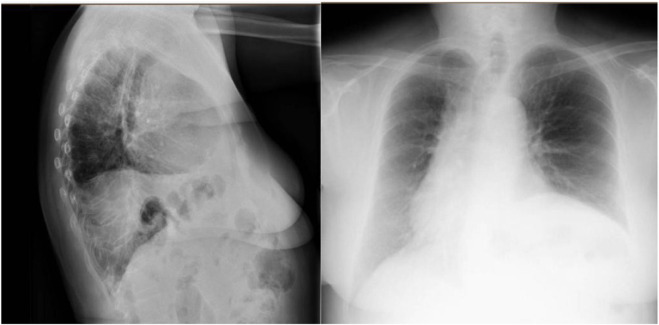
Postoperative image showing decreased—but not complete resolution of—left hemidiaphragm elevation compared to the preoperative state.

At a six-month follow-up, the patient reported a significant improvement in her quality of life, with improved respiratory function and digestive symptoms. At 12 months, the patient remained asymptomatic and without recurrence. The follow-up postoperative X-ray showing decreased—but not complete resolution of—left hemidiaphragm elevation compared to the preoperative state, which did not show an elevated diaphragm (Figure 4).

### Case 3

A 45-year-old man (body mass index 30 kg/m^2^) was evaluated for mild haemoptysis, moderate exertional dyspnoea, episodes of central chest tightness, and exercise intolerance. After being evaluated by the pulmonology department, he was diagnosed with idiopathic left diaphragmatic relaxation and referred for thoracic surgery. His medical history included being a former smoker for 2 months, asthmatic bronchitis in childhood, and admission to the emergency department in November 2022 for extensive deep vein thrombosis in the right lower extremity and acute pulmonary thromboembolism in the inferior lobar pulmonary artery and left basal segmental pulmonary arteries. Physical examination was normal.

A chest CT scan showed left lung volume loss due to a large diaphragmatic elevation with intra-abdominal visceral contents in the chest and a right-sided mediastinal shift (Supplementary Figure 1). Preoperative preparation focused on prehabilitation with respiratory physiotherapy. Given the size of the defect, adjuvant therapy was performed with botulinum toxin injection following the Smoot technique [14], without complications.

Four weeks later, diaphragmatic plication was performed with a non-absorbable barbed suture without the need for reinforcing mesh using video-assisted thoracoscopy. A pleural drain and an epidural catheter were placed for pain control. The patient was extubated in the operating room and transferred to the postoperative recovery unit, where he remained for 24 h, hemodynamically stable, eupnoeic, and without the need for supplemental oxygen. Intra-abdominal pressure was monitored, with values of 13 mmHg at the end of surgery. The epidural catheter and pleural drain were removed 48 h later, and the patient was discharged on the third day.

At a six-month follow-up, respiratory function improved without dyspnoea or recurrence (Supplementary Figure 2).

## Discussion

Diaphragmatic paralysis and eventration represent a complex surgical challenge, and several studies have evaluated diaphragmatic plication as a standard intervention.

Reviews and series in adults and children have consistently shown improvements in respiratory function, including increased forced vital capacity and reduced dyspnoea, as well as relief of symptoms such as orthopnoea and exercise intolerance following plication [1–3], [15]. However, complications such as abdominal compartment syndrome, although rare, have been reported in cases of diaphragmatic paralysis or large diaphragmatic eventrations [4], highlighting the need for careful perioperative planning. In parallel, the use of botulinum toxin type A as a preoperative adjunct has been extensively studied in the context of large ventral hernias and abdominal wall reconstruction. Multiple studies and systematic reviews have demonstrated that preoperative botulinum toxin A increases abdominal wall compliance, reduces tension during closure, facilitates the reduction of herniated contents, and, in some series, decreases postoperative complications such as seroma, infection, or hernia recurrence [5–12]. Specifically, volumetric analyses and clinical measurements have shown improved abdominal cavity accommodation and decreased intra-abdominal pressure after toxin administration [6, 9].

Evidence specifically addressing the use of botulinum toxin A prior to diaphragmatic plication is extremely limited. In isolated reports, such as the use in Morgagni hernias [16], botulinum toxin A facilitated abdominal wall relaxation, allowing for tension-free closure and reducing the risk of postoperative complications. Similarly, in giant hiatal hernias with loss of domain [17, 18], preoperative toxin injection improved diaphragmatic mobility and abdominal cavity accommodation, contributing to successful surgical reduction and repair without major adverse events. These findings suggest potential benefits, but the evidence is limited to case reports and small series, leaving a significant gap in systematic data for complex diaphragmatic eventrations.

To our knowledge, no previous study has systematically described the use of botulinum toxin type A as a prehabilitation strategy prior to diaphragmatic plication. Our series of three patients provides new evidence supporting the safety and feasibility of this approach in complex diaphragmatic paralysis with large eventrations. In all cases, preoperative botulinum toxin administration facilitated abdominal wall relaxation, allowed for tension-free plication, and was associated with favourable postoperative outcomes, including the absence of abdominal compartment syndrome and sustained clinical and radiological stability at 12 months in two patients. These findings suggest that the benefits previously observed in abdominal wall reconstruction may be extended to thoracic surgery, offering a novel strategy to optimize surgical conditions and patient outcomes in this underexplored field.

When comparing these findings with the present series, there is consistency in the effectiveness of botulinum toxin type A in improving preoperative abdominal and thoracic dynamics, reducing postoperative risks, and enhancing patient quality of life. However, it is important to note that most existing studies consist of case reports or small series, which limits the generalizability of their findings. Additionally, it would be of interest to assess the quantitative impact of botulinum toxin type A on intra-abdominal pressure, postoperative respiratory function, and objective markers of quality of life, which would enable the establishment of stronger and standardized recommendations for its use in thoracic surgery.

The present study has some limitations, such as its retrospective nature, the small number of patients included, short follow-up, and lack of a comparison group. However, this series contributes to the growth of evidence supporting the use of botulinum toxin type A as an adjunctive tool in prehabilitation before complex diaphragmatic eventrations repair.

In conclusion, although this is a novel and still underexplored indication, the preoperative administration of botulinum toxin type A as part of a prehabilitation protocol in patients with complex diaphragmatic paralysis appears to be a safe and effective strategy and may represent a significant advancement in the management of these conditions. Larger prospective studies are needed to establish standardized protocols and to validate these findings in broader populations with long-term follow-up in order to draw definitive conclusions.

## Data Availability

The raw data supporting the conclusions of this article will be made available by the authors, without undue reservation.

## References

[1] Groth SS Andrade RS . Diaphragm Plication for Eventration or Paralysis: A Review of the Literature. Ann Thorac Surg (2010) 89(6):S2146–50. 10.1016/j.athoracsur.2010.03.021 20493999

[2] Visouli AN Mpakas A Zarogoulidis P Machairiotis N Stylianaki A Katsikogiannis N Video Assisted Thoracoscopic Plication of the Left Hemidiaphragm in Symptomatic Eventration in Adulthood. J Thorac Dis (2012) 4(Suppl. 1):6–16. 10.3978/j.issn.2072-1439.2012.s001 23304437 PMC3537423

[3] Moreno-Galarraga L Bardaji C Herranz Aguirre M Viguria N . Diaphragmatic Pathology in Children: Not Always an Easy Diagnosis. Pediatr Emerg Care (2021) 37(11):e767–e768. 10.1097/PEC.0000000000001765 30829839

[4] Phadnis J Pilling JE Evans TW Goldstraw P . Abdominal Compartment Syndrome: A Rare Complication of Plication of the Diaphragm. Ann Thorac Surg (2006) 82(1):334–6. 10.1016/j.athoracsur.2005.08.054 16798249

[5] Van Rooijen MMJ Yurtkap Y Allaeys M Ibrahim N Berrevoet F Lange JF . Fascial Closure in Giant Ventral Hernias After Preoperative Botulinum Toxin A and Progressive Pneumoperitoneum: A Systematic Review and Meta-Analysis. Surgery (2021) 170(3):769–76. 10.1016/j.surg.2021.03.027 33888320

[6] Espinosa-de-Los-Monteros A Meza-Medina CA Lopez-Zamora Z Solis-Reyna RA Carrillo-Vidales J . Effects of Botulinum Toxin in Two Indicators of Loss of Domain and Hernia Size Among Patients with Large Ventral Hernias. World J Surg (2024) 48(4):881–6. 10.1002/wjs.12112 38415896

[7] Yurtkap Y van Rooijen MMJ Roels S Bosmans JML Uyttebroek O Lange JF Implementing Preoperative Botulinum Toxin A and Progressive Pneumoperitoneum Through the Use of an Algorithm in Giant Ventral Hernia Repair. Hernia (2021) 25(2):389–98. 10.1007/s10029-020-02226-2 32495050

[8] Deerenberg EB Elhage SA Shao JM Lopez R Raible RJ Kercher KW The Effects of Preoperative Botulinum Toxin A Injection on Abdominal Wall Reconstruction. J Surg Res (2021) 260:251–8. 10.1016/j.jss.2020.10.028 33360691

[9] Amaral PHF Macret JZ Dias ERM . Volumetry After Botulinum Toxin A: The Impact on Abdominal Wall Compliance and Endotracheal Pressure. Hernia (2024) 28(1):53–61. 10.1007/s10029-023-02848-2 37563426

[10] Dias ERM Rondini GZ Amaral PHF Macret JZ Carvalho JPV Pivetta LGA Systematic Review and Meta-Analysis of the Pre-Operative Application of Botulinum Toxin for Ventral Hernia Repair. Hernia (2023) 27(4):807–18. 10.1007/s10029-023-02816-w 37329437

[11] Elstner KE Read JW Saunders J Cosman PH Rodriguez-Acevedo O Jacombs ASW Selective Muscle Botulinum Toxin A Component Paralysis in Complex Ventral Hernia Repair. Hernia (2020) 24(2):287–93. 10.1007/s10029-019-01939-3 30949893

[12] Nielsen MØ Bjerg J Dorfelt A Jørgensen LN Jensen KK . Short-Term Safety of Preoperative Administration of Botulinum Toxin A for the Treatment of Large Ventral Hernia with Loss of Domain. Hernia (2020) 24(2):295–9. 10.1007/s10029-019-01957-1 31041556

[13] Sabbagh C Dumont F Fuks D Yzet T Verhaeghe P Regimbeau JM . Progressive Preoperative Pneumoperitoneum Preparation for Large Incisional Hernia Repair. Hernia (2012). 10.1007/s10029-011-0849-2 21773758

[14] Smoot D Zielinski M Jenkins D Schiller H . Botox A Injection for Pain After Laparoscopic Ventral Hernia: A Case Report. Pain Med (2011) 12:1121–3. 10.1111/j.1526-4637.2011.01147.x 21668748

[15] Beshay M Abdel BM Kösek V Vordemvenne T Mertzlufft F Schulte am Esch J . Minimally-Invasive Diaphragmatic Plication in Patients with Unilateral Diaphragmatic Paralysis. J Clin Med (2023) 12(16):5301. 10.3390/jcm12165301 37629343 PMC10455218

[16] Barber Millet S Carreño Saenz O de Juan Burgueño M Carbonell Tatay F . Empleo De Toxina Botulínica En Pared Abdominal Como Tratamiento Previo a La Reparación Quirúrgica De Una Hernia De Morgagni Gigante. Rev Hispanoam Hernia (2015) 3(2):65–9. 10.1016/j.rehah.2015.02.002

[17] Henriques CD Rodrigues EF Carvalho L Pereira AM Nora M . Adjuvant Botulinum Toxin Type A on the Management of Giant Hiatal Hernia: A Case Report. Cureus (2024) 16(2):e53836. 10.7759/cureus.53836 38465052 PMC10924647

[18] Nachtergaele S Khalil H Martre P Baste JM Roussel E . Area of Focus in 3D Volumetry and Botulinum Toxin A Injection for Giant Diaphragmatic Hernia with Loss of Domain: A Case Report with Video Illustration. J Abdom Wall Surg (2024) 3:13448. 10.3389/jaws.2024.13448 39310670 PMC11412849

