# Supplementary material for: Preoperative Botulinum Toxin for Complex Diaphragmatic Paralysis: A Case Series
Source: J Abdom Wall Surg. 2026 Jan 7;4:14476. doi: 10.3389/jaws.2025.14476 (PMC12819334; doi:10.3389/jaws.2025.14476)
Supplement: Supplementary file 1 [file Supplementaryfile1.docx]

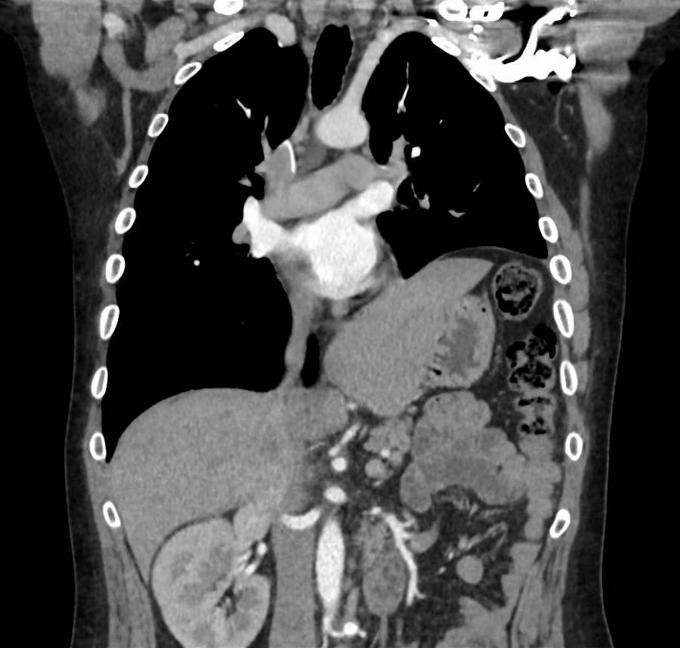

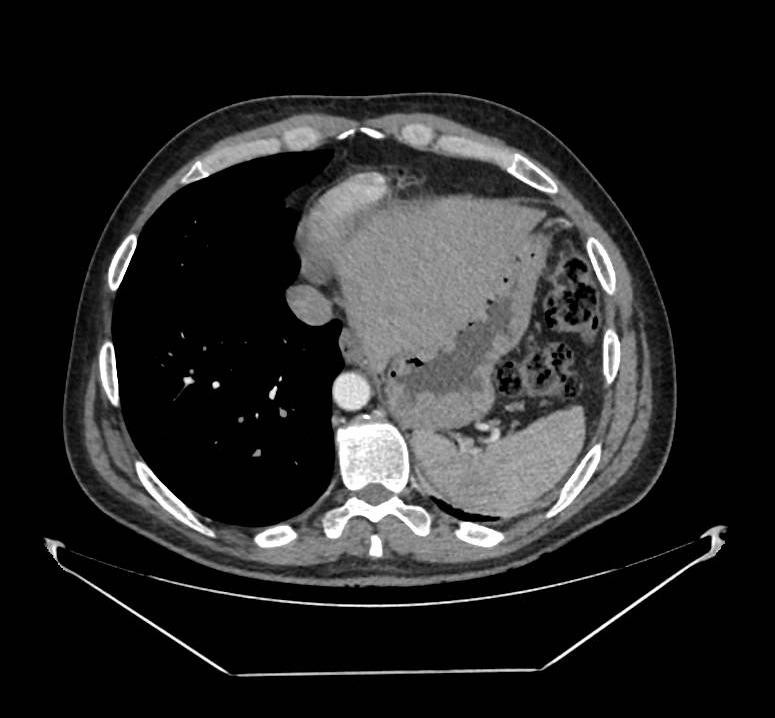


**Supplementary Figure 1.** Chest CT scan showed left lung volume loss due to a large diaphragmatic elevation with intra-abdominal visceral contents in the chest and a right-sided mediastinal shift


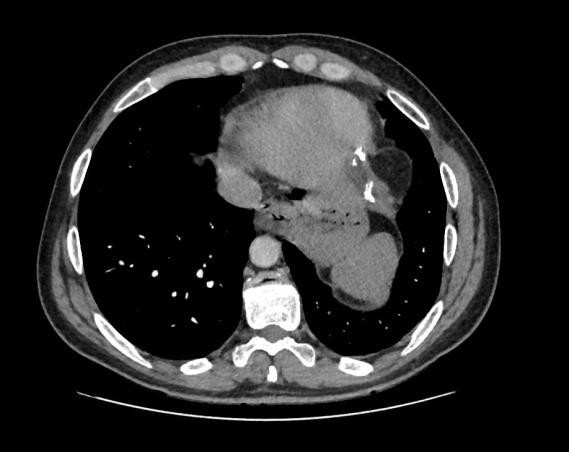

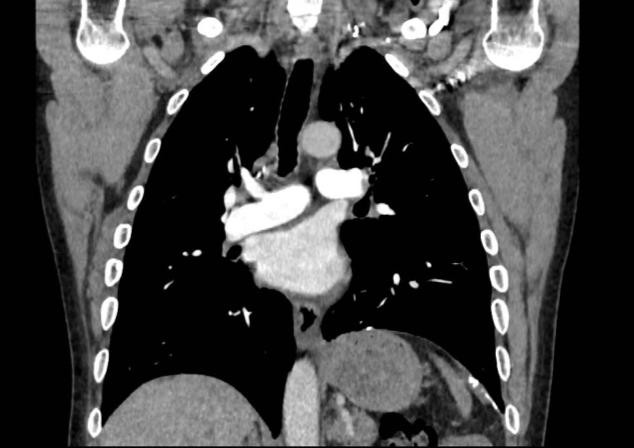


**Supplementary Figure 2.** Control CT Postoperative showing decreased—but not complete resolution of—left hemidiaphragm elevation compared to preoperative state.
